# Psoriasis complicated with arsenical keratosis and cutaneous squamous cell carcinoma: a case report

**DOI:** 10.3389/fonc.2024.1415444

**Published:** 2024-10-01

**Authors:** Qianwei Liu, Bailin Chen, Yanping Bai, Jie Zhang, Zhirong Qi

**Affiliations:** ^1^ Graduate School, Beijing University of Chinese Medicine, Beijing, China; ^2^ Department of Dermatology, National Center for Integrated Traditional Chinese and Western Medicine, China Japan Friendship Hospital, Beijing, China; ^3^ Library, Chengdu University of Traditional Chinese Medicine, Chengdu, Sichuan, China; ^4^ Department of Oncology of Integrative Chinese and Western Medicine, National Center for Integrated Traditional Chinese and Western Medicine, China-Japan Friendship Hospital, Beijing, China

**Keywords:** psoriasis, arsenical keratosis, squamous cell carcinoma, case report, malignant transformation

## Abstract

Rare cases of arsenical keratosis are attributed to the ingestion of arsenic-containing traditional Chinese medicines for conditions such as psoriasis. Arsenic is a potent carcinogen, and squamous cell carcinoma is known to develop in arsenical keratosis. A 51-year-old male patient with a 30-year history of psoriasis and a history of arsenic poisoning presented with suppuration, ulceration, and pain one and a half years after trauma to the right thumb. These symptoms had recurred after wound debridement, lesion resection, and pedicle flap transplantation. Histopathological examination of the skin lesions had suggested squamous cell carcinoma, and subsequent PET-CT examination had shown proliferation and enlargement of lymph nodes. Following right forearm amputation and radiofrequency ablation, additional lumps had been observed, but the patient had declined further surgery. Physical examination showed palpable enlarged axillary lymph nodes, which was confirmed by ultrasound. After three cycles of first-line immunotherapy with toripalimab combined with albumin paclitaxel and cisplatin chemotherapy, masses in the right upper arm were reduced. This case highlights the risks of arsenic-containing medicines used for treating psoriasis. Attention should be paid to the use of standardized treatments in psoriasis, as well as the probability of malignant transformation in arsenical keratosis.

## Introduction

Arsenical keratosis is a cutaneous manifestation of chronic arsenic poisoning, with rare cases attributed to the ingestion of traditional Chinese medicines containing realgar (xionghuang) for conditions such as asthma and psoriasis (1). Studies have identified arsenic as a potent carcinogen that can induce genetic replication, recombination, and amplification, which potentially activates viral elements and leads to both benign and malignant tumors of the skin and internal organs. The development of tumors in arsenical keratosis is predominantly characterized by Bowen’s disease and squamous cell carcinoma (2). This case report presents an exceptionally rare instance of a middle-aged patient with a history of psoriasis who developed arsenical keratosis and subsequently squamous cell carcinoma following a cutting injury.

## Case presentation

**Figure 1:**
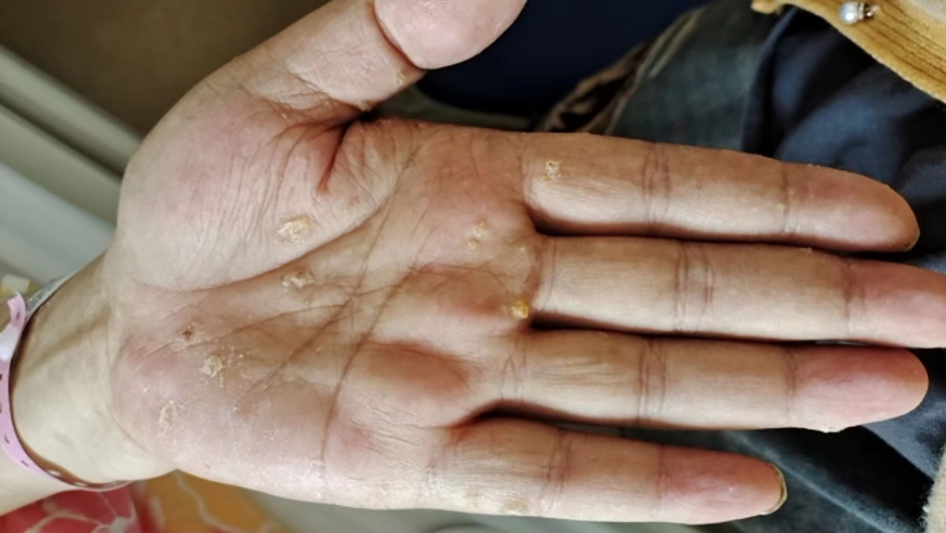
Left hand. Partial view of hyperkeratosis and verrucous hyperplasia of the palm with a diameter of 0.2–0.4 cm.

A 51-year-old man presented to the Department of Integrated Traditional Chinese and Western Medicine Oncology of our hospital with a history of nearly 30 years of psoriasis on 25 June 2023 due to “abscess, ulceration, and pain after trauma of the right thumb for one and a half years.” The patient was treated in Peking University First Hospital, and the skin lesions of the palm were diagnosed as arsenical keratosis (**Figure 1**). The patient was considered to have chronic arsenic poisoning caused by previous treatment of psoriasis without the guidance of physicians. He reported oral administration of realgar (with As_4_S_4_ as the major component) for at least 25 years.

**Figure 2:**
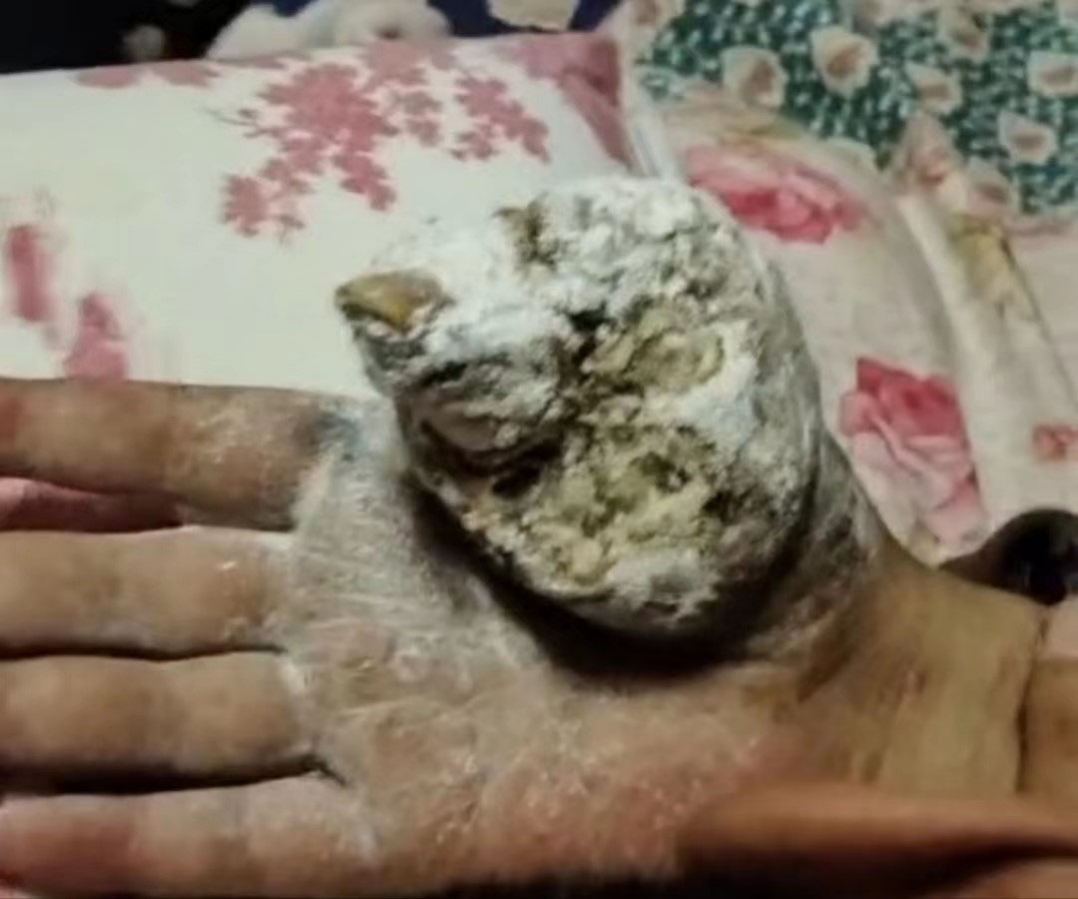
The right thumb was ulcerated, with sarcoma, pain, and odor.

**Figure 3:**
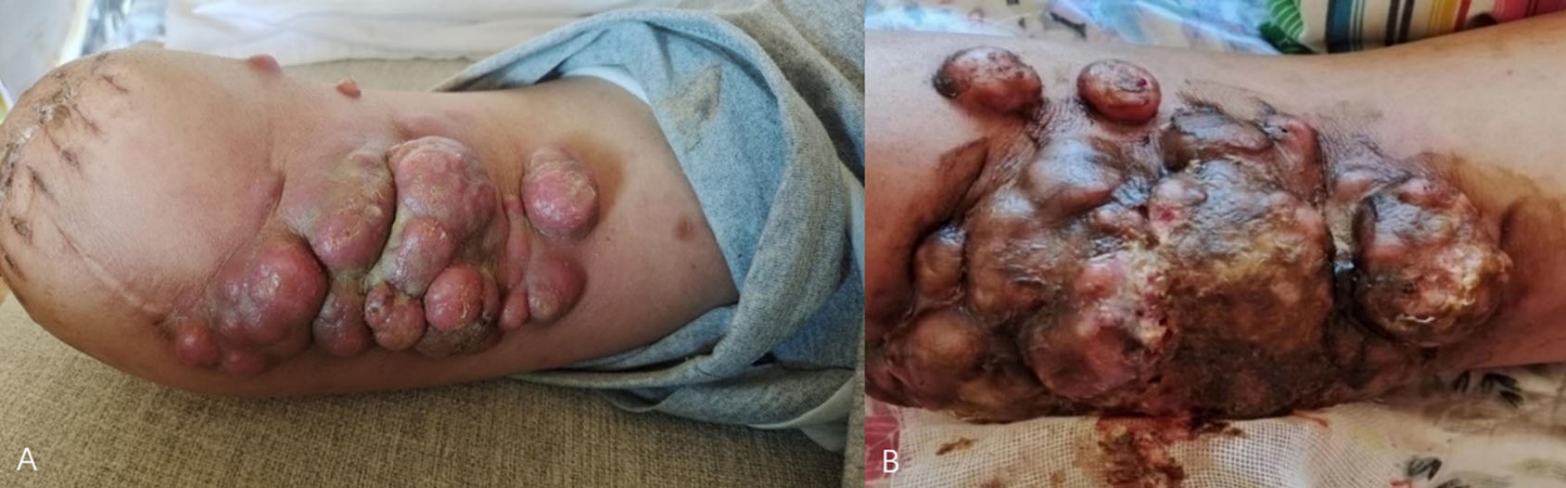
Three months after surgery, several lumps appeared on the medial side of the right upper arm. On the largest lump, a yellow secretion was observed on the skin lesion **(A)**. Four months after surgery, several lumps exhibited progressive enlargement, with masses experiencing spontaneous rupture that yielded a purulent, malodorous discharge **(B)**.

History of present illness: In January 2022, the patient was admitted to a local hospital due to suppuration and ulceration after his right thumb had been cut by a knife. Symptomatic treatment such as dressing change was given. Six months later, the wound recurred, and the patient returned to the local hospital for expanded wound debridement on 22 July 2022. On 2 August 2022, the patient underwent debridement of the right thumb + resection of soft tissue lesions + transplantation of a dorsal island pedicle flap from the index finger in the local hospital. A postoperative pathological examination showed moderately to poorly differentiated squamous cell carcinoma, but detailed histopathology images are not available. Three months after the operation, the transplanted flap had not survived, and the local tissue had gradually become ulcerated. No radiotherapy or chemotherapy was performed. The area of skin lesions gradually expanded, and the right thumb was ulcerated, with sarcoma, pain, and odor (**Figure 2**). PET-CT examination on 3 April 2023, showed the following: (1) a mass of soft tissue in the right thumb area and adjacent palmar and dorsal areas with increased proliferation; (2) several enlarged lymph nodes in the right axilla with increased proliferation; and (3) several small subcutaneous lymph nodes on the anterior side of the right upper arm, with slightly increased proliferation. On 7 April 2023, the patient underwent amputation of the right forearm and radiofrequency ablation in the local hospital. The postoperative pathological diagnosis was differentiated squamous cell carcinoma of the right hand, and the immunohistochemical results were as follows: P40 (+), CK5/6 (+), EGFR (+), P16 (−), Ki-67 (+, 30%), and PD-L1 (22C3) (combined positive score of approximately 15). One month after the amputation of the right forearm, a huge mass was found in the stump skin of the right arm, a mass was found in the skin of the medial side of the upper arm, the surface was uneven, and a huge mass was detected in the right axilla. Afterward, several lumps appeared on the medial side of the right upper arm, and these gradually increased in number. On the largest lump, a yellow secretion was observed on the skin lesion. The local hospital recommended further resection and postoperative radiotherapy and chemotherapy. The patient and his family members refused surgery because he had undergone two operations, and the tumor grew rapidly after the operation. Four months after surgery, several lumps exhibited progressive enlargement, with masses experiencing spontaneous rupture that yielded a purulent, malodorous discharge (**Figure 3**). The patient declined microbiological examination of the exudate. The patient has explicitly denied any family history of cancer or psoriasis, which indicated no known hereditary predisposition to these conditions within his lineage.

Physical examination revealed palpable enlarged lymph nodes in the right and left axillae, but the rest of the physical examination was unremarkable.

Skin condition: lumps of different sizes could be seen on the medial side of the upper arm, with obvious protrusion of the skin surface and an uneven surface. Pink granulation and a yellow or black crust could be seen. Pus could be seen under the crust, with a foul odor, overflow of yellowish-white pus, and tenderness, and the crust easily ruptured and bled on contact.

**Figure 4:**
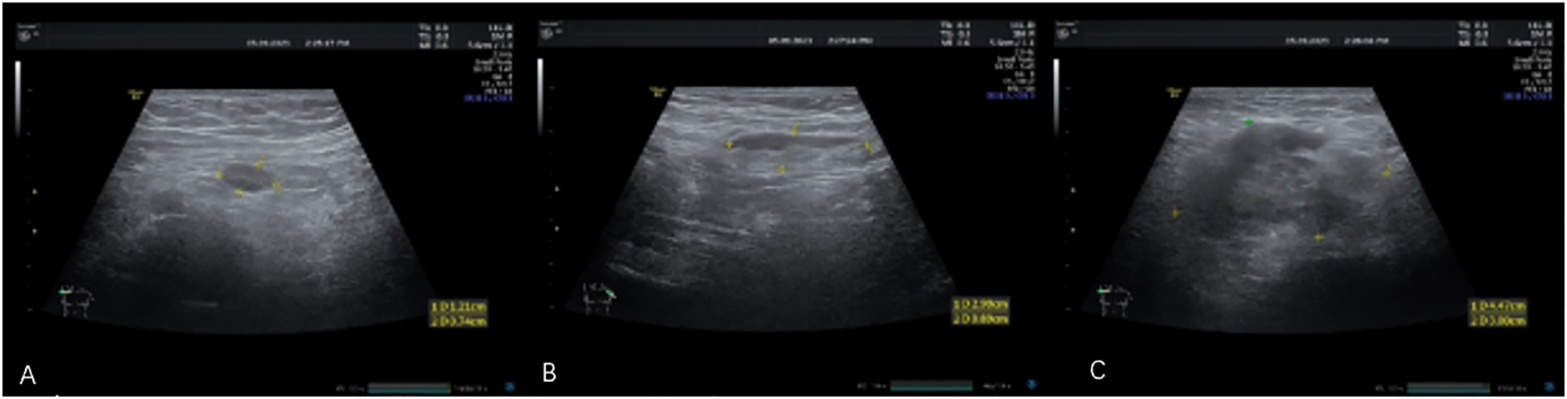
Axillary lymph node ultrasound revealed several hypoechoic bilateral lymph nodes in the axillary regions. The largest node on the right had dimensions of 1.2 × 0.7 cm **(A)**, and the largest node on the left had dimensions of 2.9 × 0.9 cm **(B)**, with indistinct architecture. Color Doppler flow imaging (CDFI) showed no definite signals of blood flow. In addition, a hypoechoic mass with ill-defined margins was observed in the right axilla. This mass had dimensions of approximately 4.4 × 3.0 × 4.0 cm **(C)**, with an irregular shape and heterogeneous internal echoes. CDFI did not demonstrate significant signals of blood flow within this mass.

Auxiliary examination: Blood routine testing: total white blood cells 10.36 × 10^9^/L ↑, total neutrophils 8.57 × 10^9^/L ↑, neutrophil percentage 82.7% ↑, lymphocyte percentage 10.8% ↓, eosinophil percentage 0.2% ↓, hemoglobin 115 g/L ↓, serum thyroid-stimulating hormone 5.58 μIU/mL ↑, serum CA72-4 7.08 U/mL ↑, progastrin-releasing peptide 111.8 pg/mL ↑, and squamous cell carcinoma antigen 19.5 ng/mL ↑. Axillary lymph node ultrasound revealed several hypoechoic bilateral lymph nodes in the axillary regions (**Figure 4**).

Diagnosis: (1) moderately differentiated squamous cell carcinoma of the skin; and (2) psoriasis vulgaris.

Treatment: From 26 June 2023 to 11 September 2023, the patient completed three cycles of first-line immunotherapy with toripalimab combined with albumin paclitaxel and cisplatin chemotherapy. The specific regimen was albumin paclitaxel 200 mg d1, d8 + cisplatin 40 mg d1–d3 + tislelizumab 240 mg d1, q21d.

After treatment, the mass in the right upper arm was significantly reduced, and the patient requested that immunotherapy be postponed. The patient is still under follow-up.

## Discussion

Arsenic is a potent carcinogen, and the World Health Organization defines chronic arsenic poisoning as “a chronic health condition arising from prolonged ingestion of arsenic above the safe dose for at least 6 months, usually manifested by characteristic skin lesions of melanosis and keratosis, occurring alone or in combination, with or without the involvement of internal organs” (1).

Arsenical keratosis is an important cutaneous manifestation of chronic arsenic poisoning, and it is also a precancerous disease (3). Some widely recognized and documented sources of arsenic exposure include arsenic-contaminated groundwater/food crops, pharmaceuticals, occupational exposure, and tobacco (2). At present, sporadic cases in China are mainly related to the use of arsenic-containing traditional Chinese medicines for the treatment of psoriasis and asthma. The main clinical manifestations include keratotic papules symmetrically distributed on both palms and soles. Typical skin lesions are corn-shaped and light yellow with rough surfaces and are slightly depressed in the center and often fused into larger masses. Hyperpigmented spots on unexposed areas such as the trunk and limbs are interspersed with hypopigmented spots (4). Histopathological examination shows that arsenical keratosis is characterized by prominent dense hyperkeratosis, parakeratosis, and acanthosis. Papillomatosis and vacuolated keratinocytes may also be present (2). The disease is easily misdiagnosed as simple hyperkeratosis, hyperpigmentation disease, etc. The diagnosis requires a careful medical history; detection of arsenic in blood, urine, and hair; and histopathological examination of the skin, if necessary, to determine whether the skin lesions are cancerous. For the treatment of patients with psoriasis with arsenical keratosis, discontinuation of arsenic therapy is recommended. In addition, it is recommended to use DMPS (2,3-dimercaptopropane-1-sulfonate sodium) to chelate arsenic to form nontoxic sulfhydryl compounds, which is beneficial for the elimination of arsenic compounds. Some drugs, such as tretinoin, retinol, nicotinamide, and selenium, may help to alleviate keratosis (5).

At present, the mechanism of arsenic-induced carcinogenesis is not clear. Potential mechanisms include direct DNA damage, influences on various cellular DNA repair mechanisms, oxidative and nitrosative stress caused by reactive oxygen species and reactive nitrogen species, respectively, and damage to immune functioning via various pathways (6). For patients with suspected skin cancer, pathological examination is still the gold standard for diagnosis. Once a diagnosis of Bowen’s disease, squamous cell carcinoma, or basal cell carcinoma is made, Mobitz’s procedure is recommended, followed by close follow-up every 3 months and regular ultrasound of the lymph nodes and abdomen. Because the palms and soles of the feet are frequent sites of arsenical keratosis, skin grafting is necessary, considering the difficulty of suturing the incision and repeated pathological analysis. For patients with severe skin lesions on the palms and soles, carbon dioxide laser treatment combined with aminolevulinic acid photodynamic therapy may be helpful for relief of symptoms.

## Limitations

In this study, we merely provided clinical data to support the diagnosis. However, we could not provide evidence of long-term arsenic exposure based on nail or hair samples. In addition, we faced the limitation that the histopathological report, which was conducted at an external hospital, was not available in its entirety. We were only provided with the diagnostic conclusion without the accompanying images, which would have otherwise enhanced the comprehensiveness of our case analysis. The patient declined microbiological examination, which resulted in a lack of microbiological results. This limitation prevents us from offering a complete assessment of the infectious etiology that might be associated with the patient’s condition. An additional limitation of this case report was the patient’s refusal to undergo further surgical treatment due to financial constraints. This decision has implications for the progression of his condition and the potential therapeutic outcomes, which could not be fully explored in our study due to this constraint.

## Conclusions

This case suggests that clinicians should promote the teaching of standardized treatments for patients with psoriasis to avoid iatrogenic injury. Patients who have used or are using arsenic-containing drugs to treat psoriasis should be aware of the risks of these drugs. Patients who have already developed arsenical keratosis should pay attention to reducing the probability of malignant transformation and take corresponding measures. Once lesions are cancerous, surgical treatment should be adopted.

## Data Availability

The original contributions presented in the study are included in the article/supplementary material. Further inquiries can be directed to the corresponding author.
